# Interannual Variation in Carbon Sequestration Depends Mainly on the Carbon Uptake Period in Two Croplands on the North China Plain

**DOI:** 10.1371/journal.pone.0110021

**Published:** 2014-10-14

**Authors:** Xueyan Bao, Xuefa Wen, Xiaomin Sun, Fenghua Zhao, Yuying Wang

**Affiliations:** 1 Key Laboratory of Ecosystem Network Observation and Modeling, Institute of Geographic Sciences and Natural Resources Research, Chinese Academy of Sciences, Beijing, China; 2 University of Chinese Academy of Sciences, Beijing, China; 3 Center for Agricultural Resources Research, Institute of Genetics and Developmental Biology, Chinese Academy of Sciences, Shijiazhuang, China; Tennessee State University, United States of America

## Abstract

Interannual variation in plant phenology can lead to major modifications in the interannual variation of net ecosystem production (NEP) and net biome production (NBP) as a result of recent climate change in croplands. Continuous measurements of carbon flux using the eddy covariance technique were conducted in two winter wheat and summer maize double-cropped croplands during 2003–2012 in Yucheng and during 2007–2012 in Luancheng on the North China Plain. Our results showed that the difference between the NEP and the NBP, i.e., the crop economic yield, was conservative even though the NEP and the NBP for both sites exhibited marked fluctuations during the years of observation. A significant and positive relationship was found between the annual carbon uptake period (CUP) and the NEP as well as the NBP. The NEP and the NBP would increase by 14.8±5.2 and 14.7±6.6 g C m^−2^ yr^−1^, respectively, if one CUP-day was extended. A positive relationship also existed between the CUP and the NEP as well as the NBP for winter wheat and summer maize, respectively. The annual air temperature, through its negative effect on the start date of the CUP, determined the length of the CUP. The spring temperature was the main indirect factor controlling the annual carbon sequestration when a one-season crop (winter wheat) was considered. Thus, global warming can be expected to extend the length of the CUP and thus increase carbon sequestration in croplands.

## Introduction

The potential of cropland to mitigate the greenhouse effect has received much attention in recent years [1]. Cropland can serve as an accumulator of carbon and can sequester approximately 50–100 Tg C each year [2]. The North China Plain, which is the largest agricultural production center in China, has an area of 3×10^5^ km^2^ and supports the production of food for over 300 million people [3]. This plain is a large area planted in winter wheat and summer maize double-cropping systems and supplies more than 50% of China's wheat and 33% of its maize [4]. Owing to its large area, this agricultural center has great potential to store carbon and reduce the emissions of greenhouse gases. Describing the strength of the carbon sink or source in this area will aid in the assessment of the regional greenhouse gas balance, in the development and validation of models that simulate a crop's carbon cycle and in the evaluation of remotely sensed data [5]. Long-term continuous measurements of carbon flux using eddy covariance technology is a practical method to assess the carbon balance and can sufficiently reflect the interannual variations in carbon exchange, which would provide scientists with valuable information for understanding the response of ecosystem processes to climate change [6].

There is an on-going debate regarding whether cropland acts as a carbon sink or as a carbon source [7]. According to published studies, the long-term variation in the ecosystem carbon exchange is usually described by the standard deviation of annual flux sum or the coefficient of variations (i.e. the ratio of standard deviation and mean value of annual flux sum) [8], [9]. In agricultural systems, annual carbon sequestration is represented by the net biome carbon production (NBP) but not the net ecosystem carbon production (NEP) [10]. The NBP in cropland is mainly consisted of three parts: the carbon absorbed by plants as NEP, carbon inputs such as the application of fertilizer or pesticide and carbon outputs resulting from harvesting and machinery [11]. Compared with the amount of carbon lost through harvesting, other forms of carbon input or emission constitute a small fraction of the NBP [12]. Owing to the large amount of carbon stored in grain, the NBP has been reported as being close to zero (i.e., carbon neutral) [13]–[17], greater than 100 g C m^−2^ y^−1^ (i.e., a carbon sink) [4], [12], [18], [19] or significantly less than zero (i.e., a carbon source) [12].

Although most previous studies that examined the seasonal variations mechanisms in agroecosystem carbon exchange were based on less than 3 years of datasets [1], [20]–[24], more recent researches have been conducted on crops to interpret the interannual variations in carbon sequestration using long-term observations of eddy covariance data [17], [25], [26]. These studies indicated that the interannual variations in agroecosystem carbon fluxes can easily be explained based on the annual variations in environmental factors such as temperature [17] and precipitation [14]. Moreover, climate factors are strong indicators of spatial patterns in terrestrial ecosystem carbon fluxes [27], [28]

Vegetation phenology, which aims to study the timing and length of the growing season in terrestrial ecosystems and their relationship with the climate, has become a meaningful earth systems science [29]. Plant phenology has been shown to be affected by climate changes and, in turn, influences ecosystem processes such as the carbon cycle [30]. As an intermediary of ecosystems and climate change, variations in plant phenology can be used to interpret the seasonal and interannual variations in carbon fluxes and their relationship with climate change [31]. The effects of variation in plant phenology on the annual net ecosystem carbon exchange have been reported in different ecosystems at local [32] and continental scales [33]. These studies indicated that the annual net ecosystem carbon storage was significantly related to the growing season length (GSL) [34]–[40], the CUP [37], [41] and the leaf area index (LAI) [31]. According to published studies, the impact of the interaction between climate change and the abovementioned typical land surface phenological variables on annual ecosystem carbon sequestration is variable [9], [31], [32], [42], [43]. Most studies have focused on one season per year for forest and grassland ecosystems; it has been suggested that climate factors, especially air temperature, would affect the starting [44] or the ending date of the growing season [43] or the time interval in days between the gross ecosystem production (GEP) and the CUP [42] and would ultimately influence the annual ecosystem carbon budget.

Many studies have been conducted in the critical area of the North China Plain to explore temporal carbon budget variations based on continuous eddy covariance datasets. Li et al. (2006) [19] interpreted the mechanisms underlying the seasonal variations in crop carbon exchange using 2 years of flux data. Shen et al. (2013) [31] quantified the carbon fluxes of crops under different water conditions based on 4 years of data. Tong *et al.* (2014) [45] studied the light response characteristics of crop carbon exchange using 4 years of flux data. In the present study, continuous measurements of carbon flux using eddy covariance were conducted in two winter wheat/summer maize double-cropped croplands from 2003 to 2012 in Yucheng and from 2007 to 2012 in Luancheng. Our objectives were to (1) quantify the interannual variability in the NEP and the NBP and (2) investigate the environmental and biotic controls over the interannual variations in the NEP and the NBP.

## Materials and Methods

### 1. Ethics Statement

The two sites in this study are maintained by the Institute of Geographic Sciences and Natural Resources Research, Chinese Academy of Sciences and the Institute of Genetics and Developmental Biology, Chinese Academy of Sciences. Both areas are practice bases for researchers at the Chinese Academy of Sciences. All necessary permits were obtained for the fields study. The field study did not involve endangered or protected species.

### 2. Site description

This study was carried out at the Yucheng Comprehensive Experimental Station (36°57′N, 116°38′E; elevation of 23.4 m) in Shandong Province and at the Luancheng Comprehensive Experimental Station (37°50′N, 114°40′E; elevation of 50.1 m) in Hebei Province. Both sites are located on the North China Plain and are part of the Chinese Ecosystem Research Network (CERN) and the ChinaFLUX network. The climate conditions of the two sites are relatively similar. These sites are within the East Asia monsoon region characterized by a semi-humid and warm temperate climate. The mean annual temperature and precipitation are 13.1°C and 528 mm, respectively, in Yucheng (average values from 1975 to 2005) [22] and 12.8°C and 485 mm, respectively, in Luancheng (average values from 1990 to 2008) [31]. Winter wheat (*Triticum aestivum* L.) and summer maize (*Zea mays* L.) are cultivated in rotation each year. The typical growing season extends from mid-October to the next mid-June for winter wheat and from late June to October for summer maize. In Yucheng, the sowing and harvest date of winter wheat ranged from 10 October 2004 to 29 October in 2005 and from 7 June 2007 to 16 June 2010, respectively. In Lucheng, the sowing date and harvest date of winter wheat ranged from 7 October 2011 to 19 October 2007 and from 11 June 2008 to 17 June 2009, respectively. The sowing date of summer maize varied from 13 June 2005 to 22 June 2011 in Yucheng and from 6 June 2010 to 19 June 2008 in Luancheng. The summer maize was harvested from 18 September 2006 to 14 October 2005 in Yucheng and from 23 September 2009 to 2 October 2008 and in Luancheng. Nitrogen fertilizer was applied to the farmlands after the planting date. The crops were irrigated using well water during the reviving and jointing stages of winter wheat and during planting or the jointing stage of summer maize. The crops were irrigated with 100–150 mm of water on each irrigation date [31]. In Yucheng, the ground water table varies from 1.5 to 3.5 m with an average of 2.5 m, the soil in the depth of 1∼20 cm is consist of clay loam, silt loam and sand loam with the portion of 22.1%, 65.1%, 12.8%, respectively. In Luancheng, the soil in the depth of 1∼25 cm is predominated by sand loam. The bulk density of the soil is 1.3 gcm^−3^ [56] and 1.4 gcm^−3^ in Yucheng and Luancheng, respectively [57].

### 3. Flux and meteorological measurements

Similar monitoring instrumentation and data collection methods were used in Yucheng and Luancheng. The eddy covariance system consisted of a three-dimensional sonic anemometer (Model CSAT 3, Campbell Scientific Inc., USA) to monitor fluctuations in vertical wind velocity and an open-path and fast-response infrared gas analyzer (Model LI-7500, Li-Cor Inc., USA) to monitor the fluctuations in the concentrations of CO_2_ and water vapor. The flux towers were located in the center of the crop fields at both sites, and the fetch was greater than 200 m. The eddy covariance (EC) systems were set on the tower at a height of 2.8 m in Yucheng and a height of 3.5 m in Luancheng. All of the raw data were collected continuously at 10 Hz using a data logger (Model CR5000, Campell Scientific Inc., USA), and the 30 min mean data were outputted.

A suite of micrometeorological sensors was mounted above the canopy and in the soil [19], [26], [46]. These sensors provided half-hourly measurements of net radiation (Model CNR-1, Kipp and Zonen, Netherlands), photosynthetic photon flux density (LI190SB, Li-Cor Inc. USA), air temperature and relative humidity (Model HMP45C, Vaisala Inc., Helsinki, Finland), wind speed and direction (Model AR-100, Vector Instruments, UK), soil water content (Model CS615-L, Campbell Scientific), soil temperature (TCAV, Campbell Scientific, USA), soil heat flux (Model AR-100, Vector Instruments, UK) and precipitation (Model 52203, RM Young Inc., Traverse City, MI, USA). All the data were recorded using data loggers (Model CR23XTD, Campbell Scientific Inc., USA).

### 4. Flux calculation, gap filling and partitioning

The half-hourly turbulent CO_2_ flux (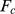, µmol CO_2_ m^−2^ s^−1^) above the crop canopy, i.e., the net ecosystem carbon exchange (NEE, µmol C m^−2^ s^−1^), can be calculated from the covariance between the vertical wind velocity (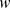, m s^−1^) fluctuation and the CO_2_ density fluctuation (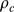, µmol CO_2_ m^−3^) according to Reynolds' decomposition rule: 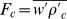
(1)where the primes denote the turbulent fluctuations (departures from the mean) and the overbar indicates a time-averaged mean (30 min). Several procedures have been performed to correct the 30 min mean output data. First, to satisfy the hypothesis of the eddy covariance technology, a tilt correction was necessary for the systems. The tilt error caused by the nonparallel fixation of the anemometers with the field surface was corrected by double rotation to align 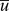 with the mean wind direction, forcing 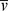 to 0 and the mean vertical velocity (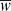) to 0 [47]. Second, as with all measuring instruments, the eddy covariance system is not able to completely capture the true turbulence when sufficiently high and low frequencies occur, which results in the loss of information compared with ideal conditions. These missing flux data can arise for several reasons, such as an inadequate sensor frequency response, separation of the instruments (particularly the sonic anemometer and the infrared gas analyzer), line averaging and distributed sampling [48]. Therefore, the spectral correction method of Eugster and Senn (1995) [48] was indispensable in this study to compensate for the missing raw covariances. Finally, the Webb-Pearman-Leuning (WPL) correction was applied to correct the error caused by the transfer of heat and water vapor because fluctuations in temperature and humidity can cause variation in the density of trace gases [49].

During monitoring, there were occurrences of sensor malfunction, rain events, instrument maintenance and power failures, which resulted in missing or anomalous values of the flux and the meteorological data. For the meteorological data, we deleted the data over the normal range, and the data which has deviated more than 1.96 standard deviation of a time series from the mean value. Then, we applied a linear interpolation method to fill in the missing data that had a time interval less than 2 h and the mean diurnal variation (MDV) method to fill in the missing data that had a time interval longer than 2 h [50]. In addition, missing flux data resulted from the exclusion of these data under low-turbulence conditions at night when the wind friction velocity (u_*_) was less than the threshold value. The threshold value was determined from the relationship between u_*_ and the nighttime NEE. The total missing nighttime flux data is about 36%±3% (the mean and standard deviation of ten years) and 43%±2% (the mean and standard deviation of five years) of annual total flux data in Yucheng and Lucheng, respectively. The total missing daytime flux data was about 14%±2% and 24%±7% of annual total flux data in Yucheng and Luancheng, respectively. Linear interpolation was also used to fill the flux data gaps shorter than 2 h. The marginal distribution sampling (MDS) method was used to fill the flux data gaps longer than 2 h, by which the missing flux data could be “looked up” on the basis of environmental factors such as the photosynthetic photon flux density and the temperature correlations with the missing data [51].

The NEE is the sum of the gross ecosystem carbon exchange (GEE) and the ecosystem respiration (RE). The daytime GEE could not be obtained directly from the EC systems, but it could be obtained as an estimate of RE. In general, the nighttime RE is equal to the nighttime NEE because the nighttime GEE is zero. By using the nighttime flux data under high turbulence, a regression model (the LT model) described by Lloyd and Taylor (1994) [52] was used to fit the model parameters 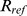 and E_0_, and then the new model was used to estimate the daytime RE. The Lloyd and Taylor model is as follows:
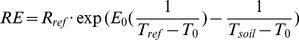
(2)where 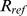 denotes the ecosystem respiration rate at a reference temperature (T*_ref_*  = 10°C), *E*_0_ is a parameter associated with the activation energy and determines the temperature sensitivity of RE and T_0_ is a constant temperature parameter (−46.02°C) [52].

Because the crop LAI changed dramatically, the RE was affected by the temperature and the crop phenology. To consider the effects of phenology on the calculation of RE, the long-term dataset was divided into a series of short subperiods (the window was 15 days), which were shifted by 5 days, i.e., the overlap between adjacent windows was 10 days. The regression of the LT model was performed separately for each subperiod [51].

In our analysis, we used the net ecosystem production (NEP) rather than the NEE (NEP = -NEE) and the gross ecosystem production (GEP) rather than the GEE (GEP = -GEE). The sign of the NEP is positive when CO_2_ is transported from the atmosphere to the ecosystem and is negative when CO_2_ is transported in the opposite direction.

### 5. Calculations of the plant phenological factors

In farmland, the growing season length (GSL) is usually defined as the total number of days from the sowing date to the harvest date [53]. The carbon uptake period (CUP) could be calculated only by using the eddy covariance data because the CUP was defined as the number of days when the ecosystem acts a carbon sink [32]. We determined the CUP as the number of days when the agroecosystem is a net carbon sink during the growing season [13], and we calculated this value from the smoothed moving average of the NEP [32]. The leaf area index (LAI, m^2^ m^−2^) of the crop was measured using an area meter (LI-3100, Li-Cor, NE, USA) 1–4 times per month during the growing season.

### 6. Calculation of the net biome production (NBP)

In agroecosystems, the net biome production (NBP) can be obtained from the following equation: 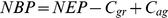
(3)where *C_gr_* refers to the carbon content of the grains, which is a product of the harvest, and *C_ag_* refers to the other forms of carbon transfer, including diesel combustion, the application of fertilizer and pesticide and the transport of inputs and grains. *C_gr_* was estimated from the following:
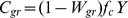
(4)where *W_gr_* is the grain water content, i.e., 0.140 for wheat and 0.155 for maize; *f*_c_ is the fraction of the carbon in the grain, i.e., 0.45 for wheat and 0.447 for maize; and *Y* is the crop yield [20]. It is worth noting that Bernacchi *et al.* (2005) [7] indicated that the *C_ag_* accounted for approximately 7% and 10% of the NBP in a soybean crop and a corn crop, respectively, when all of the carbon transformation events were accounted for. In fact, according to the experimental records of the Chinese Ecosystem Research Network (CERN), the workers in Yucheng and Luancheng manually weed the fields rather than apply pesticide. In addition, they also use limited nitrogen fertilizer (only 1–2 times per growing season), thereby releasing very little CO_2_ to the atmosphere. The carbon released by fuel combustion and machinery transportation only occurs during the harvest. As a result, we inferred that the *C_ag_* would account for a small fraction of the NBP and we ignored it when calculating the NBP in this study.

### 7. Data statistics and analysis

To determine the dominant factor controlling the interannual variations in carbon exchange, several procedures were adopted to analyze the dataset in this study. The initial variables used to interpret the variation in the mechanisms of the carbon flux were environmental factors that included the air temperature, the soil temperature at a depth of 5 cm, the precipitation, the photosynthetic photon flux density (PPFD) and phenological factors such as the GSL, the LAI and the CUP. First, we applied the stepwise method in the multiple regression by PASW statistic 18 software (2010, ver18.0; SPSS Inc) to select the significant independent variables (p<0.05). The independent variable(s), which entered multiple regression models, would have significant effect on the dependent variables. And then, the path analysis was performed to interpret the relationship between the independent variables and their direct and indirect effects on the interannual variations in the NEP and NBP. The path analysis were conducted by Amos (2003, ver5.0; SPSS Inc).

## Results

### 1. Interannual variations in the meteorological factors and the leaf area index

**Figure 1:**
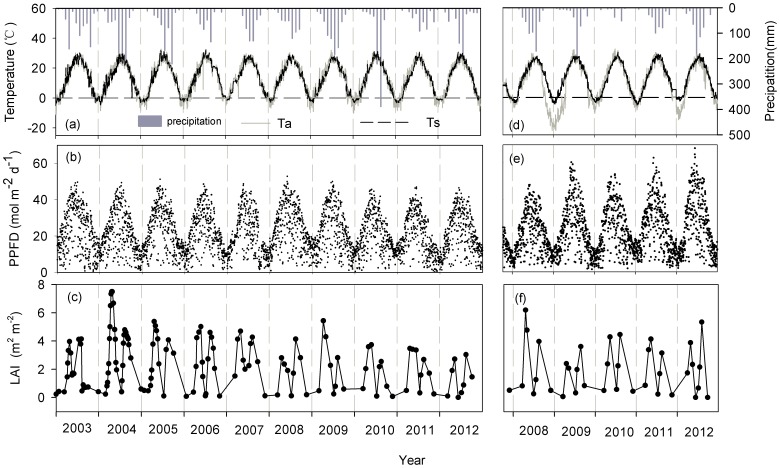
The seasonal and interannual variation in the meteorological and biotic variables. The variables include daily average air temperature (black solid line), the daily average soil temperature at a depth of 5 cm (black dotted line) and the cumulative monthly precipitation (vertical black bar) in Yucheng YC (a) and Luancheng LC (d), the cumulative daily photosynthetic photon flux density (PPFD) in YC (b) and LC (e), and the leaf area index data (LAI) measured in YC (c) and LC (f). Data from 2003 to 2012 in Yucheng (YC) and from 2007 to 2012 in Luancheng (LC) are presented.

Fig. 1 shows the seasonal and interannual variations in the air temperature, soil temperature at a depth of 5 cm, precipitation, photosynthetic photon flux density (PPFD) and leaf area index (LAI) in Yucheng and Luancheng. Table 1 also lists the annual values of these variables. The seasonal variation in the daily mean air temperature exhibited a single-peak curve, and the maximum and minimum values occurred from July to August and from November to the next January, respectively. The annual air temperature ranged from 11.5 to 13.9°C (12.9±0.7°C, mean±standard deviation) and 8.0 to 12.7°C (10.4±2.2°C) in Yucheng and Luancheng, respectively. The seasonal trend in the soil temperature was similar to the seasonal trend in the air temperature, but the daily soil and air temperature were not equal to each other. The annual soil temperature was close to the annual air temperature in Yucheng, the annual soil temperature was higher than the air temperature (13.1±0.4°C) in Luancheng. Because the straw residue of the summer maize remained in the field after harvesting, the rate of the decrease in the air temperature was more dramatic than the soil temperature when extreme cold weather occurred during the years of 2008–2009 and 2011–2012 in Luancheng. The asynchronous changes in the air and the soil temperature resulted in different decline amplitudes in Luancheng. Generally, the precipitation frequency in the second half of the year was higher than that in the first half of the year. The annual precipitation was 528.3±197.2 mm and 399.5±170.0 mm in Yucheng and Luancheng, respectively. The seasonal variation in the PPFD also exhibited a single peak, with the strongest radiation in April or May each year. The total annual PPFD was 8250.6±535.6 mol m^−2^ in Luancheng, which was higher than that in Yucheng (7670.1±369.0 mol m^−2^). The LAI is considered to be an indicator of crop development (Fig. 1c). The LAI was much lower during the germination stage and then gradually increased to reach a maximum during the bloom stage. The mean annual LAI ranged from 0.7 to 2.4 m^2^ m^−2^ (1.4±0.5 m^2^ m^−2^) in Yucheng and from 1.0 to 1.9 m^2^ m^−2^ (1.5±0.4 m^2^ m^−2^) in Luancheng.

**Table 1:** The mean annual air temperature (MAT, °C), the soil temperature at a depth of 5 cm (Ts, °C), the photosynthetic photon flux density (PPFD, mol/m^2^), the precipitation (P, mm), the leaf area index (LAI, m^2^/m^2^), the growing season length (GSL, days), the carbon uptake period (CUP, days) and the start date of the carbon uptake period (CUP_start-date_) in Yucheng (YC) and Luancheng (LC) in the North China Plain.

Season	Sites	MAT	Ts	PPFD	P	LAI	GSL	CUP	CUP _start-date_
Whole year	YC	12.9±0.7	12.9±0.8	7670.1±369.0	528.3±197.2	1.4±0.5	343.6±12.4	162.0±12.5	64.0±13.4
	LC	10.4±2.2	13.1±0.4	8250.6±535.6	399.5±170.0	1.5±0.4	349.2±12.5	142.2±17.9	77.6±12.2
Winter wheat	YC	6.9±4.2	6.7±4.6	4556.0±338.2	107.6±237.8	1.2±0.5	237.4±7.9	88.9±10.6	198.1±6.0
	LC	8.3±4.9	10.8±1.5	4488.4±765.7	82.9±43.5	1.2±0.3	242.8±12.0	82.0±11.8	201.4±5.0
Summer maize	YC	25.2±0.7	25.8±0.9	2713.4±254.9	360.3±138.7	1.9±0.6	106.6±6.6	74.0±6.0	64.0±13.4
	LC	24.2±1.6	24.9±0.6	3089.5±373.9	300.6±130.5	2.3±0.4	106.4±6.6	60.0±11.6	77.6±12.2

The mean value and the standard deviation from 2003 to 2012 in YC and from 2007 to 2012 in LC are presented.

### 2. Interannual variations in carbon fluxes and net biome production (NBP)

**Figure 2:**
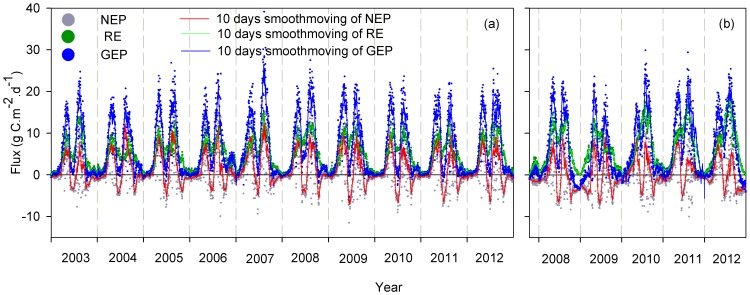
The seasonal and interannual variation in the cumulative daily fluxes. Fluxes include net ecosystem production (NEP) (gray dots), the cumulative daily ecosystem respiration (RE) (green cross), and the cumulative daily gross ecosystem production (GEP) (blue open circle). The 10-day running mean of the NEP (red line) for Yucheng (YC) (a) and Luancheng (LC) (b) on the North China Plain is also presented.

The seasonal and interannual variations in the daily NEP, GEP and RE in Yucheng and Luancheng are presented in Fig. 2. The 10-day running mean of the NEP, GEP and RE are also shown. In winter, the carbon fluxes were close to zero from the sowing stage in October to the reviving stage the next March due to the slow growth of winter wheat. The leaves photosynthesized at a low rate because of the low LAI, and there were low rates of respiration because of the low temperatures. Moreover, there were not sufficient photosynthetic substrates for autotrophic respiration. However, it is worth noting that during the winter from 2008 to 2009 in Luancheng, a portion of the wheat seed have died as the result of the low air temperature, and the reduction in photosynthesis converted the crop ecosystem to a carbon source. With an increase in the temperature in spring, the winter wheat began to grow rapidly, and the dynamics of the GEP and the RE corresponded to the changes in the LAI. The NEP became positive, and the crops started to absorb carbon. The NEP, GEP and RE had almost reached their maximum value simultaneously in late April when the winter wheat LAI was at its maximum. However, the maximum value of the RE of the winter wheat was delayed compared with the GEP in 2010 in Luancheng. The lag may be attributed to both environmental and phenological factors. The carbon fluxes decreased because of plant senescence. The seasonal dynamics of the summer maize carbon fluxes also corresponded with the seasonal trends in the LAI. Because the germination stage of summer maize is shorter than that of winter wheat, the summer maize began to absorb carbon in mid-July (approximately one month after sowing). The NEP of the summer maize increased dramatically after the germination stage and usually reached its maximum value in mid-August.

**Figure 3:**
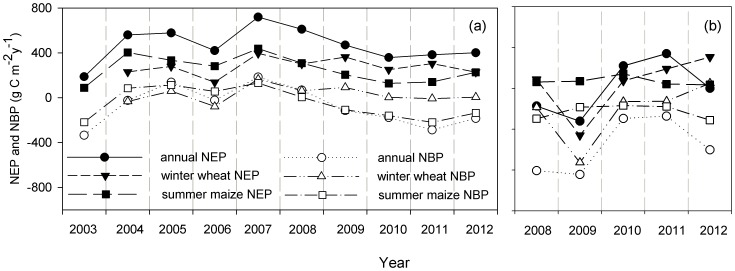
The interannual variations in annual and seasonal NEP and NBP. Interannual variations in annual NEP, annual NBP, seasonal accumulative NEP and NBP during winter wheat growing season, seasonal accumulative NEP and NBP during summer maize growing season in Yucheng (YC) (a) and Luancheng (LC) (b) on the North China Plain are presented.

The interannual variations in annual and seasonal NEP and NBP are showed in Fig.3. The amplitude of the interannual variations in the NEP and the NBP were different. The annual NEP at the Yucheng site ranged from 187.1 to 718.5 g C m^−2^ yr^−1^, with a long-term mean of 475.6±159.4 g C m^−2^ yr^−1^. The annual NEP at the Luancheng site ranged from −322.2 to 304.2 g C m^−2^ yr^−1^, with a long-term mean of 43.0±353.3 g C m^−2^ yr^−1^. The NEP in Luancheng in 2008 and 2009 was extremely low (annual NEP was −172.9 and −322.2 g C m^−2^ yr^−1^, respectively) compared with other years. This difference was attributed to the low air temperature, i.e., the mean air temperature of the last two months of 2008 and the first three months of 2009 was only −12.9°C in Luancheng, which was significantly lower than the mean air temperature during the same period in other years, e.g., 2.0°C during 2007–2008, −0.4°C during 2009–2010, 1.5°C during 2010–2011 and −2.2°C during 2011–2012. The low air temperature prohibited plant photosynthesis, but the RE continued due to the warmer soil conditions during late 2008 and the beginning of 2009 (the relationship between the RE and the soil temperature during the entire winter wheat growing season during 2008–2009 in Luancheng can be described using an exponential increase equation, i.e., RE = 0.08e^0.0834Ts^, R^2^ = 0.53, p<0.0001). The annual NBP ranged from −334.7 to 136.6 g C m^−2^ yr^−1^ (−76.1±174.5 g C m^−2^ yr^−1^) in Yucheng and from −844.3 to −130.1 g C m^−2^ yr^−1^ (−564.1±272.2 g C m^−2^ yr^−1^) in Luancheng.

When the winter wheat and summer maize were studied separately, our results showed that the seasonal accumulative NEP during winter wheat growing season ranged from 137.0 to 394.1 g C m^−2^ yr^−1^ (276.0±76.8 g C m^−2^ yr^−1^) in Yucheng and from −460.0 to 306.0 g C m^−2^ yr^−1^ (39.5±294.5 g C m^−2^ yr^−1^) in Luancheng. The accumulative NBP during winter wheat growing season ranged from −79.6 to 164.9 g C m^−2^ yr^−1^ (28.9±73.1 g C m^−2^ yr^−1^) in Yucheng and from −726.9 to 50.8 g C m^−2^ yr^−1^ (−223.7±295.0 g C m^−2^ yr^−1^) in Luancheng. The seasonal accumulative NEP during summer maize growing season ranged from 87.9 to 436.8 g C m^−2^ yr^−1^ (254.6±118.1 g C m^−2^ yr^−1^) in Yucheng and from 34.6 to 142.4 g C m^−2^ yr^−1^ (70.2±42.9 g C m^−2^ yr^−1^) in Luancheng. The accumulative NBP during summer maize growing season ranged from −219.9 to 130.8 g C m^−2^ yr^−1^ (−45.9±138.0 g C m^−2^ yr^−1^) in Yucheng and from −313 to −169.3 g C m^−2^ yr^−1^ (−228.8±70.3 g C m^−2^ yr^−1^) in Luancheng (Fig.3).

### 3. Factors affecting the interannual variation in carbon sequestration

**Figure 4:**
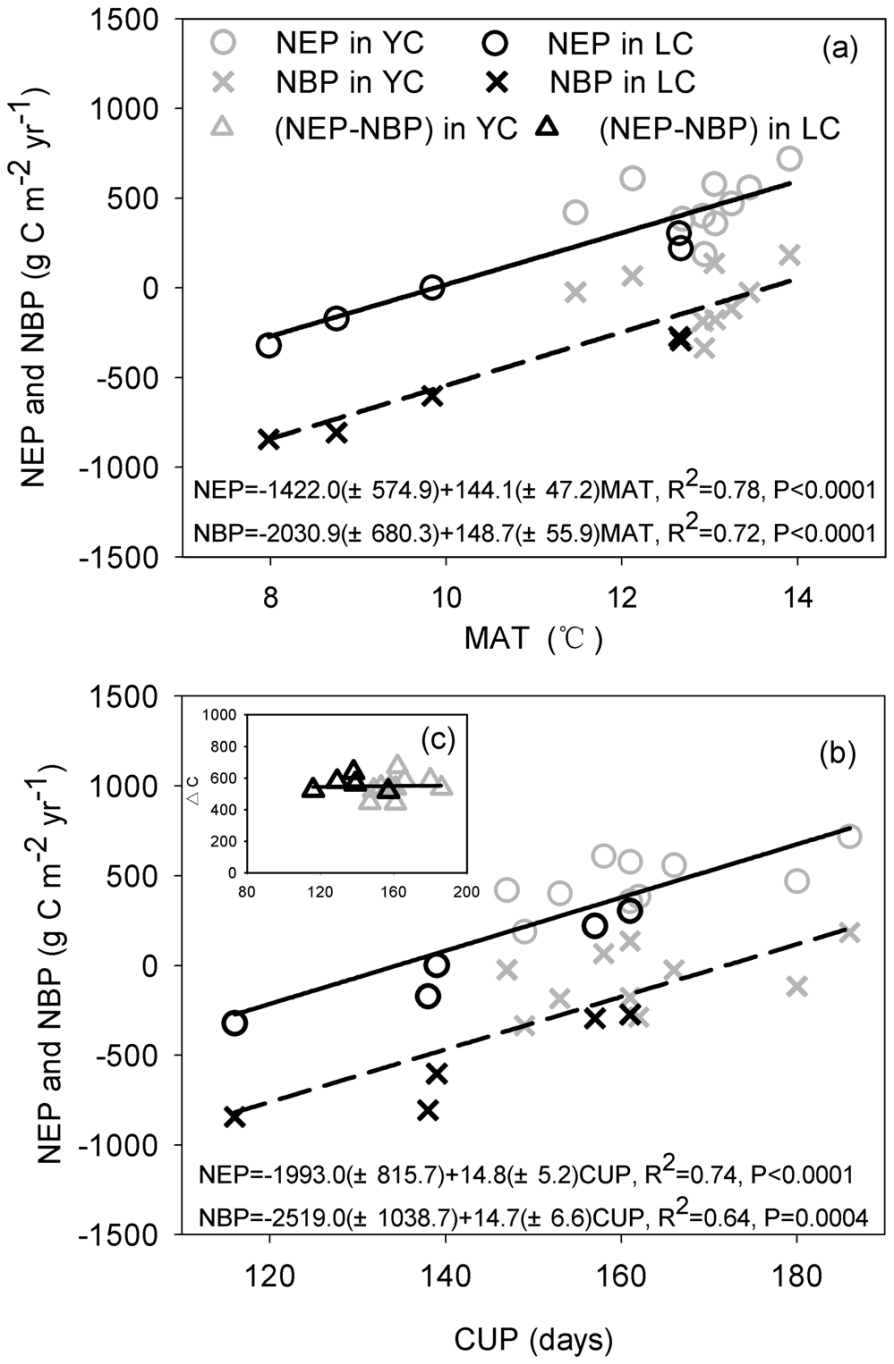
The dependence of annual carbon sequestation on meteorological and biotic variables. The dependence of the net ecosystem production (NEP) and the net biome production (NBP) on (a) the mean annual air temperature (MAT) and (b) the total C uptake period (CUP) in Yucheng (YC) and Luancheng (LC) and (c) the dependence of the difference between the NBP and the NEP (△C), i.e., the annual crop yield, on the CUP in YC and LC on the North China Plain.

**Figure 5:**
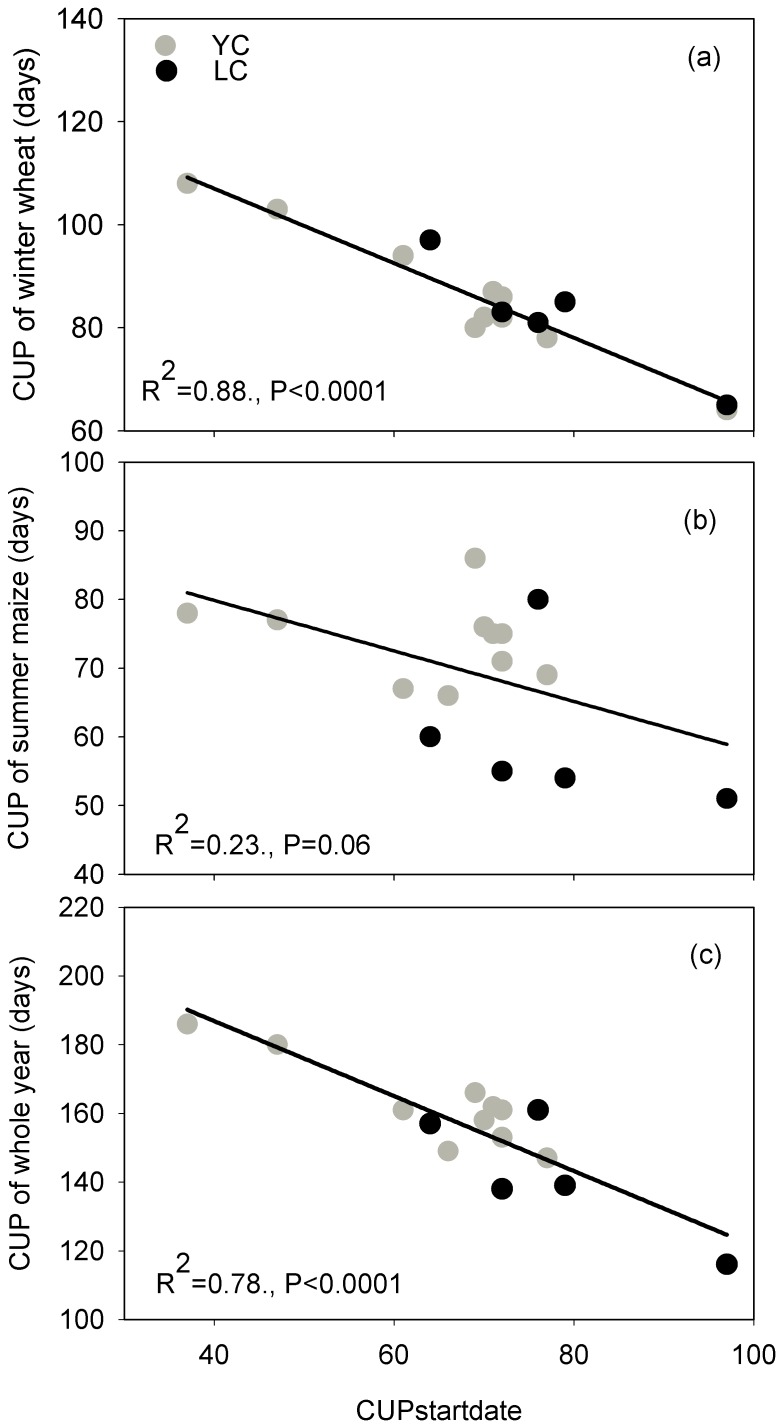
The relationships between the CUP and the start date of the CUP (CUP_start-date_). The relationships between the CUP of (a) winter wheat, (b) summer maize, (c) the whole year and the start date of the CUP (CUP_start-date_) on the North China Plain. CUP is the abbreviation of C uptake period. The start date of the CUP represents the beginning of the CUP. The solid line is the regression line.

**Figure 6:**
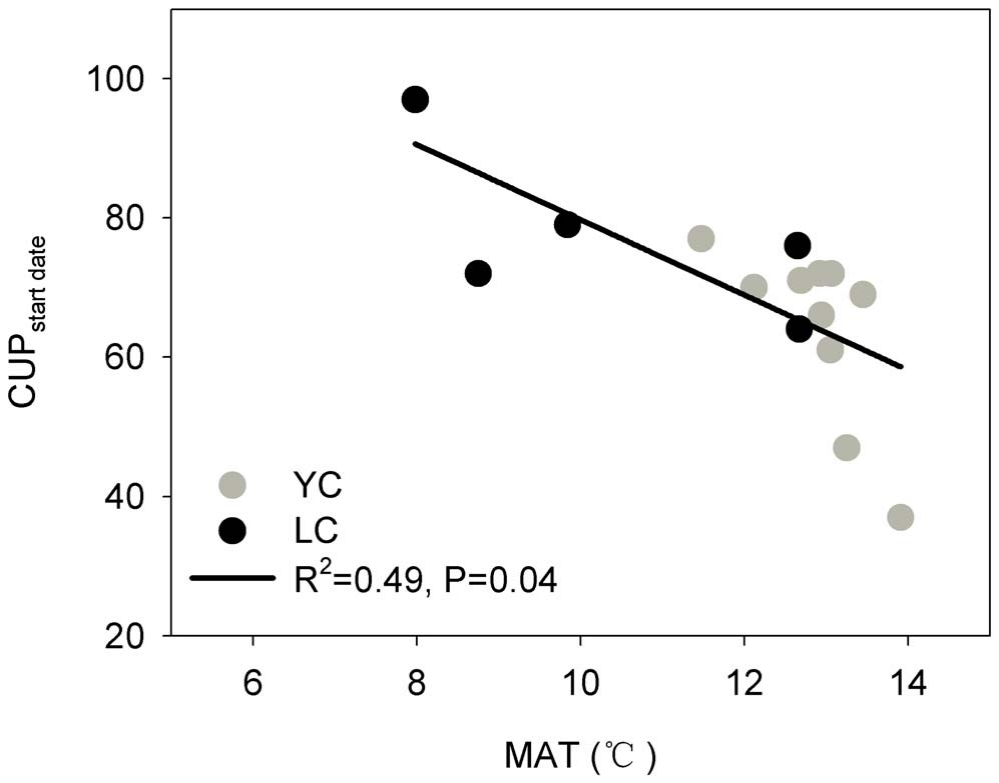
Factor affecting the start date of carbon uptake period (CUP _start-date_). The relationship between the mean annual air temperature (MAT) and the start date of the C uptake period (CUP_start-date_) in Yucheng (YC) (a) and Lucheng (LC) on the North China Plain. The solid line is the regression line.

Fig. 4 shows the dependence of the annual NEP and NBP on the mean annual air temperature (MAT) and the annual carbon uptake period (CUP). Table 1 also lists the annual values of the growing season length (GSL), the CUP and the LAI. The interannual variations in the NEP and NBP were significantly correlated with both the MAT (R^2^ = 0.78 for the NEP and 0.72 for the NBP, p<0.001) (Fig. 4a) and the annual total CUP (R^2^ = 0.74 for the NEP and 0.64 for the NBP, p<0.001) (Fig. 4b) when the two sites were combined. The starting date of CUP (CUP_start-date_) had a strongly effect on annual CUP (R^2^ = 0.78, p<0.0001 for two sites combined; R^2^ = 0.78, p<0.01 in Yucheng; R^2^ = 0.67, p<0.0001 in Lucheng) (Fig. 5), and the CUP_start-date_ was significantly affected by the MAT (R^2^ = 0.49, p = 0.04 for sites combined; R^2^ = 0.49, p = 0.025 in Yucheng; R^2^ = 0.047, p = 0.21 in Lucheng) (Fig. 6). No relationship was observed between the annual NEP or the NBP and the GSL or between the NEP or the NBP and the mean annual LAI. The annual total GSL was very close to the length of an entire year because of the short duration (only several days) of the fallow stage (this refers to the interval stage between the two crops). Compared with the GSL, the crop CUP experienced a large fluctuation between years. The CUP of the winter wheat ranged from 78 to 108 days (88.9±10.6 days) in Yucheng and from 64 to 97 days (82.0±11.8 days) in Luancheng. The CUP of the summer maize was shorter than that of the winter wheat, which was 74.0±6.0 days and 60.0±11.6 days in Yucheng and Luancheng, respectively. As a result, the annual total CUP, which was the sum of the CUP of the winter wheat and the CUP of the summer maize, increased from 147 to 186 days with a long-term mean of 162.3±12.5 days in Yucheng and from 116 to 161 days with a mean of 142.2±17.9 days in Luancheng. The linear regression models suggested that the NEP would increase by 144.1±47.2 g C m^−2^ yr^−1^ per additional degree Celsius and by 14.8±5.2 g C m^−2^ yr^−1^ per additional CUP day. The NBP also increased by a similar amount when one additional unit temperature was added and the CUP was extended (Fig. 4). Despite both the NEP and NBP being affected by the CUP, the difference between them, i.e., the crop yield was not obviously influenced by the CUP and was conservative over a long time scale (Fig. 4c).

**Figure 7:**
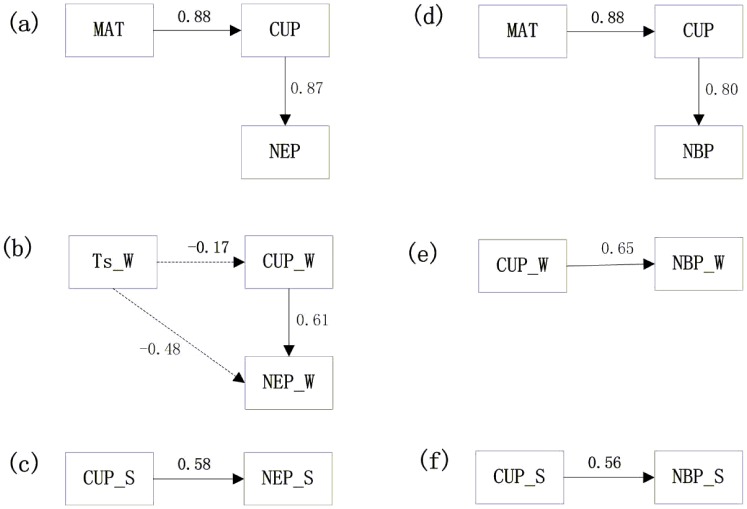
The path analysis of effect of meteorological and biotic variables on NEP or NBP. The direct and indirect effect of the mean annual air temperature (MAT), the annual total C uptake period (CUP), the soil temperature of the winter wheat (Ts_W), the C uptake period of winter wheat (CUP_W) and the C uptake period of summer maize (CUP_S) on the control of the interannual variations in (a) the annual NEP, (b) the annual NBP, (c) the seasonal NEP of winter wheat (NEP_W), (d) the seasonal NBP of winter wheat (NBP_W), (e) the seasonal NEP of summer maize (NEP_S) and (f) the seasonal NBP of summer maize (NBP_S) in Yucheng (YC) and Luancheng (LC). The solid and dashed arrows represent positive and negative correlations, respectively. The path coefficients are presented in the diagrams.

Given that the annual carbon sequestration of the crops was affected by both the MAT and the annual CUP, it was necessary to distinguish which was the main driving factor. Fig. 7 demonstrates the direct and indirect effect of the environmental and phenological factors on the control of the interannual variations in the NEP and the NBP in croplands. The annual NEP and NBP were controlled mainly by the annual CUP and had path coefficients of 0.87 and 0.80, respectively. Because of the significant relationship between the MAT and the annual CUP (the path coefficient was 0.88), the MAT had an indirect effect on the NEP and the NBP through its effect on the interannual variation in the CUP (Fig. 7a, d). This finding indicated that the annual CUP was the primary driving factor. In Yucheng, the CUP explained 40% (p = 0.049) of the interannual variation in the NEP and 19% (p = 0.21) of the variation in the NBP. In Luancheng, the values were 96% (p = 0.009) and 85% (p = 0.03), respectively. No relationship was observed between the MAT and the NEP or the NBP in Yucheng, but the MAT in Luancheng explained 95% (p = 0.004) and 99% (p<0.0001) of the interannual variation in the NEP and the NBP, respectively. The better linear relationship between MAT or CUP and NEP or NBP in Luancheng than Yucheng may result from the different soil texture and water related soil properties in both sites.

The factors controlling the interannual variation in the NEP of the winter wheat were the 5-cm depth soil temperature (Ts) of winter wheat and the winter wheat CUP, both of which were statistically significant (p<0.05). No relationship was found between the winter wheat soil temperature and the winter wheat CUP (R^2^ = 0.03, p>0.05); hence, the path coefficient was small (−0.17; the negative sign represents a negative relationship). Therefore, no indirect effect of the winter wheat soil temperature on the winter wheat NEP through the winter wheat CUP was possible. Because the path coefficient of the relationship between the winter wheat CUP and the NEP (0.61) was larger than the path coefficient of the relationship between the winter wheat Ts and the NEP (−0.48) (Fig. 7b), the CUP was the predominant factor controlling the interannual variation in the winter wheat NEP. Only the winter wheat CUP was adopted in the path analysis and this was considered the single factor that controlled the interannual variation in the winter wheat NBP (Fig. 7e). Similarly, the summer maize CUP was the only factor that controlled the summer maize NEP and the NBP (Fig. 7c and f).

## Discussion

### 1. Annual carbon budget and comparison with other terrestrial ecosystem

Our study showed that the annual NEP was 475.6±159.4 g C m^−2^ yr^−1^ and 13.0±272.5 g C m^−2^ yr^−1^ for Yucheng and Luancheng, respectively. The large standard deviation of annual NEP in Lucheng was due to the low value annual NEP in 2008 and 2009, which was −172.8 g C m^−2^ yr^−1^ and −322.2 g C m^−2^ yr^−1^ compared to other observation years (220.3 g C m^−2^ yr^−1^, 340.2 g C m^−2^ yr^−1^ and 0.71 g C m^−2^ yr^−1^ in 2010, 2011, 2012, respectively). After accounting for the grain harvest, the annual carbon sequestration (NBP) was −76.1±174.5 g C m^−2^ yr^−1^ for Yucheng and −564.0±272.2 g C m^−2^ yr^−1^ for Luancheng. Therefore, Luancheng was a stronger carbon source than Yucheng due to the low-temperature effect on the RE in Luancheng.

**Figure 8:**
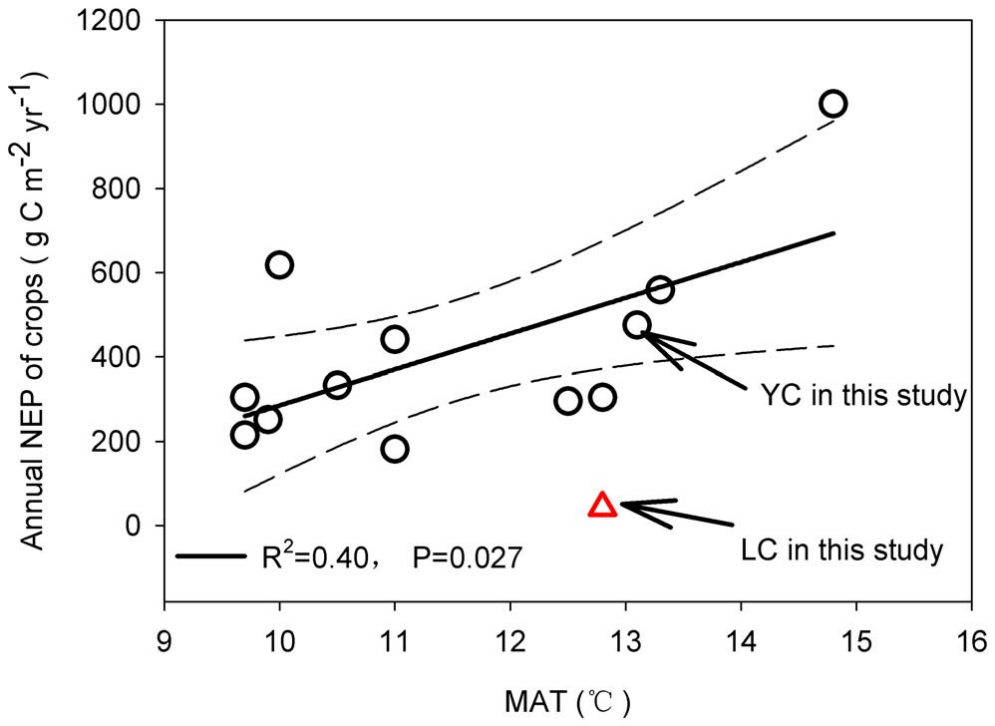
The relationship between the mean annual air temperature (MAT) and the net ecosystem production (NEP). The data was collected from literature and the dots represent annual NEP at the different sites. The solid line is the regression line, and the dotted line is the 95% band.

There are wide fluctuations in the annual carbon budget between different agroecosystems in other regions (Table 2). A series of studies have suggested that the spatial pattern of carbon flux in terrestrial ecosystems (mainly refer to forest and grassland) is driven by climate factors such as temperature or precipitation [12], [28]. Similarly, the spatial pattern of the NEP for different crop sites was also clearly explained by the mean air temperature (R^2^ = 0.40 p = 0.027) (Fig. 8). However, in the present study, the NEP in Luancheng deviated from the regression line because the crops suffered from abnormal climate conditions in late 2008 and at the beginning of 2009, as noted above, and much carbon was lost in both years. A similar result was not observed for the NBP. The combined effect of the differences in grain removal, field management and environmental conditions influenced the variations in the NBP between sites [54].

**Table 2:** Comparisons of climate condition, carbon fluxes and net biome productions among different farmlands.

Country	Site and latitude	Vegetation	MAT	P	GEP	RE	NEP	NBP	References
Belgium	Lonzee50°33′08″N, 4°44′42″E,	Sugar beet	10.0	800	—	—	610±110	—	Moureaux *et al*. 2006 [22]
Germany	Gebesee 51°06′0.13″N 10°54′51.9″E,	Winter wheat	9.7	505	—	—	185∼245	−45∼−105	Anthoni *et al*. 2004 [18]
Germany	Selhausen 50°52′0.14″N 6°26′5.58″E	Winter wheat	9.9	698	1360	1081	252	246∼201	Schmidt *et al.* 2012 [19]
France	Aurade43°54′97″N 01°10′61″E	Rapeseed	12.8	671∼724	—	—	286(±23)	≈60	Béziat *et al*. 2009 [10]
		Winter wheat					324(±20)	≈50	
		Sunflower					28(±18)	≈−110	
France	Lamasquere 43°49′65″N 01°23′69″E,	Triticale	12.5	615∼681	—	—	335(±42)	−100	Béziat *et al*. 2009 [10]
		Maize					186(±42)	−372±78	
		Winter wheat					369(±33)	161±66	
Netherland	Wageningen 51°59′28.04″N 5°38′42.44″E	Maize	10.5	803	1982	1652	333	204	Jans *et al.* 2010 [5]
USA	Illinoins 40.006°N 88.290°W	Maize	9.7	505	—	—	576.4	184	Hollinger *et al.* 2005 [14]
		Soybean			—	—	33	−94	
USA	Ponca 36°46′N 97°08′W,	Winter wheat	14.8	866	2853	1852	1001	—	Gilmanov *et al*. 2003 [65]
USA	Nebraska 41°09′54″N 96°28′35″W,	Maize	11	570	—	—	441	−13	Verma *et al*. 2005 [15]
USA	Rosemount St Paul. MN 44°10′N 93°9′W	Corn/soybean rotation(tillage)	11	956	—	—	188	−46	Baker and Griffis 2005 [21]
		Corn/soybean rotation(reduced tillage)			—	—	175	−43	
China	Yucheng 36°57′N, 116°38′E,	Winter wheat/summer maize rotation	13.1	528	2227±202	1759±122	475±159	−64±181	This study
China	Weishan 36°39′N, 116°03′E,	Winter wheat/summer maize rotation	13.3	532	1668∼2008	1135∼1423	533∼585	82	Lei and Yang 2010 [17]
China	Luangcheng N37°50′,E114°40′	Winter wheat/summer maize rotation	12.8	485	2370±476	2335±237	43±353	−519±360	This study

Climate condition includes annual mean air temperature (MAT, °C), annual precipitation (P, mm). Carbon fluxes include gross ecosystem production (GEP, gCm^−2^y^−1^), ecosystem respiration (RE, gCm^−2^y^−1^) and net ecosystem production (NEP, gCm^−2^y^−1^).

### 2. Arguments about uncertainty

There was a systemtematic uncertainty associated with choosing any algorithm for gap-filling. A series of studies have discussed the effects of gap-filling method on the sum flux [58], [59]. Moffat *et al*. (2007) [59] found that in most cases, the algorithms being used were approaching the noise limit (uncertainty) of the measurement. However, highly empirical approaches, such as marginal distribution sampling (MDS), would performed the best across a range of forested European sites. At annual scale, difference among methods were generally modest, because most calculated annual NEE integrals were within ±25 gCm^−2^y^−1^ of the mean. According to those analyses, it is possible to suggest that the uncertainty of preference of gap-filling method is relatively small when one of the highly empirical approaches is used if the gap is not too long and if the data set is large enough and have good quality to get model parameters.

Leaving crop residues (e.g., stems, leaves and roots) in the field is an important farmland management practice. The impact of crop residues on the change in seasonal carbon fluxes can be observed in Fig. 2. After the winter wheat harvest in mid-June, the RE did not decrease to zero but exhibited peaks. This pattern occurred because the suppression of the autotrophic respiration of the winter wheat resulting from the harvest was compensated for the increase in the heterotrophic respiration caused by leaving the residues in the field. Moreover, the high temperatures and the rain events in summer might also contribute to this increase in the RE. In this study, we did not estimate the carbon released from the decomposing crop residue. A series of studies have evaluated the contribution of crop residue carbon emissions to total ecosystem respiration after complete decomposition. These studies suggested that the additional emissions from the crop residues may account for a considerable part of the total carbon release. Moureaux *et al.* (2008) [23] indicated that the carbon content of winter wheat residue was 290 g C m^−2^ yr^−1^, which corresponds to approximately 30% of the RE during the cultivation period. In a three-crop rotation, the carbon content of sugar beet residue contributed approximately 40% to the next crop ecosystem RE [10]. Therefore, it is necessary to consider the effect of this important field management technique when evaluating the annual carbon sequestration of a crop and its response to climate change in future research. By not calculating the carbon emissions from the crop residue in the present study, we may have introduced an important source of uncertainty in the assessment of carbon sequestration and its response to environmental or biotic factors.

Another source of uncertainty in the analysis of carbon budget in this study may come from the neglect of C_ag_. As aforementioned above, C_ag_ usually includes the carbon emission from applying agrochemicals (fertilizer, herbicides, insecticides and fungicides), from the use of machinery and from the combustion of fuel. Many published researches have suggested the indirect carbon emission from C_ag_ [60]–[62]. Maraseni *et al*. [60] have indicated that the amount of greenhouse gas emission because of the three main farm input was large in cropping land over 30 years, and the emission amount was 17094 kg carbon dioxide equivalents (CO_2_e) for use of agrochemicals, 13272 kg CO_2_e for the use of fuel and 1910 kg CO_2_e for the farm machinery, respectively. When the site-scale research was conducted, this part of carbon emission to atmosphere would have some potential influence on assessment of annual carbon budget. Furthermore, the North China Plain is a so large area that is predominated by wheat-maize rotation system that this part of carbon emission would not be low.

### 3. The increase of carbon sequestration per extended CUP day and comparison with other ecosystems

The role of the CUP in affecting carbon sequestration has been well studied in forest and grassland systems, and these studies indicated that the annual NEP was strongly correlated with the critical phenological factor [3], [41]. Our study also suggested that the interannual variations in both the NEP and the NBP were controlled mainly by the annual CUP (Fig. 4 and Fig. 7a and d) when the two sites were combined. The annual NEP and NBP would increase by 14.8±5.2 and 14.7±6.6 g C m^−2^ yr^−1^, respectively, if one CUP-day was extended. The increment amplitudes differed from those of other ecosystems, according to published studies. In a temperate deciduous forest, the NEP increased by 5.9 g C m^−2^ yr^−1^ per extended growing season day [37]. In a boreal coniferous forest, the increase was 6.9 g C m^−2^ yr^−1^ [31]. The NEP value may be lower for grassland systems. For example, Ma *et al.* (2007) [40] reported that the NEP would increase by 2.0 g C m^−2^ yr^−1^ and 4.0 g C m^−2^ yr^−1^ per extended growing season day for a savanna and an open grassland, respectively. The increase of carbon sequestration per extended growing season day in cropland in present study is much higher than forest and grassland ecosystem. The cropland is an artificial ecosystem and usually have simple ecosystem constituent to guarantee crop economic yield, and the cropland CUP is not as flexible as forest and grassland, which have multiple plant species to maintain biological diversity. In addition, owing to the various habits of green leaves, plants can obtain their organic carbon through different paths over the same length of time. The rate of carbon uptake or carbon release is higher for a plant with a shorter growing season (<1 year), whereas a plant with a longer growing season (>1 year) usually has a lower photosynthetic rate and a lower rate of substrate decomposition [38].

### 4. Interpreting the effect of MAT on the CUP

After path analysis, we have found a clearly relationship between MAT and CUP (Fig.7) and it can be interpreted by Fig. 5 and Fig. 6. Our result shows that the length of CUP was strongly controlled by the beginning date of CUP (CUP start-date) (Fig. 5), an early starting of CUP can lead to a longer CUP. This important date would be affected by MAT significantly (Fig. 6), a higher MAT can result an early beginning of CUP. As a result, the higher MAT, through its positive effect on the CUP_start-date_, prolonged the length of the CUP and thus increased the carbon sequestration each year in the croplands.

The conclusion that a higher mean annual air temperature would result in an earlier beginning of the CUP and prolong the duration of the CUP in this study contradicts other literature. For example, Black *et al.* (2000) [46] and Welp *et al.* (2007) [55] indicated that it was a higher spring air temperature rather than a higher mean annual air temperature that led to an earlier leaf-out and an earlier start of the growing season, which ultimately increased the annual NEP. To analyze the effect of a warm spring on the interannual variation in carbon sequestration, we did not define the ‘spring period’ based on the meteorological standard division (i.e., from March to May) but rather as February to March according to Welp *et al.* (2007) [55], who noted that ‘spring’ was the period during which plants would capture significant ecosystem carbon fluxes during the early growing season. In this study, the winter wheat was sown in October but began to grow rapidly during the next February to March after a period of dormancy to acclimate to the cold environment. Therefore, we defined the spring temperature (Tsp) as the mean temperature of February and March. Our results indicated that through its significant effect on the CUP_start-date_ (R^2^ = 0.45 for Tsp vs CUP_start-date_; R^2^ = 0.49 for MAT vs CUP_start-date_), the Tsp and the MAT could explain 83% and 51%, respectively, of the interannual variation in the winter wheat NEP. Moreover, the Tsp and the MAT could explain 42% and 78%, respectively, of the interannual variation in the annual NEP. Therefore, the Tsp was the main factor controlling the annual NEP in a one-season crop (winter wheat), but it was the MAT in a two-season rotation crop that determined the annual NEP. Consequently, our results are in agreement with previous studies when we only consider winter wheat. However, when the two rotation crops are taken into account, our findings contradict the results of previous studies.

There were also studies focused on the effect of ending date of carbon uptake period on annual carbon sequestration. For example, Piao et al. (2008) [43] suggested that the ending date of CUP (CUP _end-date_) would be advanced because of warming autumn and could therefore lead to loss carbon. Wu et al. (2012) stated that the interval between the ending date of the GEP and the ending date of the CUP (i.e., the autumn interval) in forest ecosystem would affect annual carbon sequestration. However, the current study has neither found the relationship between CUP _end-date_ and annual NEP or NBP nor the relationship between the autumn interval and annual carbon sequestration, partly because the differences scales in those researches and other differences in types of ecosystems and thus in the photosynthetic pathways, in climate conditions, in ecosystem management and uncertainties in the flux data.

## Outlook

Irrigation is an important management in cropland and can impact vegetation growth, eco-physiological characteristic of plant, ecosystem ecological processes and crop production obviously [63], [64]. However, the effects of irrigation on dynamic of carbon sequestration and its relationship between environmental or biological factor can not be detected in this study because of the well and similar irrigation conditions in the two sites. There were many studies have reported the influence of water conditions on the carbon exchange in cropland. Suyker et al. (2012) found that the annual NEP was 552.0±73.0 gCm^−2^y^−1^ and 471.0±52.0 gCm^−2^y^−1^ in irrigate and rain-fed maize crop, respectively, who has also indicated that the peak value of weekly ensemble average of NEP were 2.5 mgCm^−2^s^−1^ and 2.1 mgCm^−2^s^−1^ in irrigate and rain-fed maize. Thus it can be seen that irrigation may be a source of variations of carbon flux, and, in future, how the temperature or carbon uptake period influence carbon sequestration in rain-fed field would become an interesting topic in North China Plain.

The current study further confirms the fact that the growing season length is a strong indicator of annual carbon sequestration not only in forests and grasslands but also in crop systems. However, it seems that different climate factors can affect the growing season length. Because of different physiological and ecological processes in plant leaves and canopies and their required climate conditions, it is not reasonable to state that one dominant factor that affects the annual carbon absorption in one ecosystem is the same factor in another ecosystem. Therefore, further research is needed on the interannual variation in ecosystem carbon flux in different sites and regions.

## Conclusions

In this study, the interannual variations in the carbon fluxes were presented based on eddy covariance data collected in two winter wheat/summer maize double-cropping systems from 2003 to 2012 in Yucheng and from 2007 to 2012 in Luancheng. The annual NEP was 475.6±159.4 g C m^−2^ yr^−1^ and 13.0±272.5 g C m^−2^ yr^−1^ for Yucheng and Luancheng, respectively. After accounting for the grain harvest, the annual carbon sequestration (NBP) was −76.1±174.5 g C m^−2^ yr^−1^ for Yucheng and −564.0±272.2 g C m^−2^ yr^−1^ for Luancheng. The carbon uptake period (CUP) had a strong effect on the annual NEP and NBP. The crop could gain 14.8±5.2 g C m^−2^ per CUP day on an annual basis. Our study also showed that farmland will sequester more carbon as a result of a higher MAT. The path analysis indicated that there was a significant positive correlation between the MAT and the annual NEP or NBP, which was attributed to the strong effect of the MAT on the CUP. A higher MAT could result in an earlier start date of the carbon uptake season, which would significantly affect the length of the CUP and thereby increase carbon sequestration in croplands. Moreover, the spring temperature had a strong effect on annual carbon sequestration of winter wheat, whereas it was not the determined factor control on annual carbon balance of a double-cropping agroecosystem.
